# Blood Inflammatory Markers as Predictors of Effusion Characteristics and Postoperative Hearing Outcomes in Children with Otitis Media with Effusion: A Retrospective Study

**DOI:** 10.3390/medicina61091520

**Published:** 2025-08-25

**Authors:** Amani Abdullah Almutairi, Ibrahim K. Aljabr, Zahra Saleh Alsindi, Amnah Ali Alkhawajah, Jinan Mohammed Aljasem, Mohammed Mousa Alzahrani, Abdullah Almaqhawi

**Affiliations:** 1 College of Medicine, King Faisal University, Al Hofuf P.O. Box 400, Saudi Arabia; 220007808@student.kfu.edu.sa (A.A.A.); 220025858@student.kfu.edu.sa (Z.S.A.); 220016079@student.kfu.edu.sa (A.A.A.); 220007995@student.kfu.edu.sa (J.M.A.); 2 Division of ENT, Departments of Surgery, College of Medicine, King Faisal University, Al Hofuf P.O. Box 400, Saudi Arabia; ialjabr@kfu.edu.sa; 3 College of Medicine, Al-Baha University, Albaha P.O. Box 1988, Saudi Arabia; dr.m.almousa@outlook.sa; 4 Department of Family and Community Medicine, College of Medicine, King Faisal University, Al Hofuf P.O. Box 400, Saudi Arabia

**Keywords:** otitis media with effusion, adenoid hypertrophy, neutrophil-to-lymphocyte ratio, systemic inflammation, paediatric hearing loss, tympanostomy

## Abstract

*Background and Objectives*: Otitis media with effusion (OME), frequently associated with obstructive adenoid hypertrophy (OAH), is a leading cause of paediatric hearing loss. Clinically distinguishing effusion types (serous vs. mucoid) and predicting postoperative hearing recovery are unresolved challenges. This study evaluated the utility of preoperative blood inflammatory markers in predicting effusion characteristics and short-term hearing outcomes following adenoidectomy with tympanostomy tube (TT) insertion. *Materials and Methods*: In this retrospective cohort study, 232 children under 12 years old in 2024 and undergoing adenoidectomy (with or without TT insertion) were categorised into serous OME (*n* = 42), mucoid OME (*n* = 78), and non-effusion (*n* = 112) groups. Preoperative blood sample analyses assessed neutrophil, lymphocyte, eosinophil, basophil, and platelet counts, along with derived indices, including neutrophil-to-lymphocyte ratio (NLR), platelet-to-lymphocyte ratio (PLR), eosinophil-to-basophil ratio (EBR), mean platelet volume (MPV), and systemic immune–inflammation index (SII). Hearing was evaluated at 2 weeks and 1 month postoperatively. Statistical analyses used SPSS v.28, with significance set at *p* < 0.05. *Result*: Mucoid OME patients exhibited significantly elevated neutrophil counts, platelet counts, eosinophils, NLR, and SII compared to those in serous OME and non-effusion groups (*p* < 0.05). All serous OME children achieved normal hearing by the first follow-up, whereas 15.4% of mucoid OME cases had transient mild hearing loss persisting after 2 weeks (*p* = 0.008; OR=15.97) but resolving by 1 month. Preoperative neutrophil count independently predicted delayed hearing recovery (*p* = 0.021). *Conclusions*: Systemic inflammatory markers, particularly neutrophil count, NLR, and SII, effectively differentiate mucoid OME from other effusion types and correlate with short-term hearing recovery. Neutrophil count may serve as a prognostic tool for surgical planning and patient counselling. Prospective studies are warranted to validate these findings in broader paediatric populations.

## 1. Introduction

The primary cause of conductive hearing loss in children is otitis media accompanied by effusion (OME), which is characterised by fluid accumulation in the middle ear without acute signs of infection [1]. Among the various factors which contribute to the development of OME, obstructive adenoid hypertrophy (OAH) is particularly significant as it is associated with dysfunction of the eustachian tube [2]. In the United States, approximately 2.2 million new cases of OME are diagnosed annually [3], while in Saudi Arabia, the prevalence among school-aged children is estimated to be around 7.5% [4]. OME affects most children during early childhood, with nearly 90% experiencing at least one episode before school age [5]. In school-aged populations, the prevalence of OME is reported to be around 10%, and studies have suggested an association with reduced academic performance, and speech and language acquisition, among affected children [6,7]. However, in approximately 25% of cases, it can be recurrent or persist for three months or longer, which may result in hearing loss, balance issues, challenges in receptive language development, reduced attention span, and worsened school performance [5]. Clinically, children with OME frequently present with both hearing difficulties and speech development delays [8]. For instance, a study found that over 90% of children with OME showed preoperative phonological deficits, highlighting the developmental burden of the condition [9]. Therefore, it is best to be diagnosed early and treated correctly [10]. Interventions, including adenoidectomy with or without the installation of a breathing tube, are frequently essential to restore hearing and avert long-term developmental problems [3,11]. Adenoidectomy is mostly conducted to address otitis media with effusion and obstructive sleep-disordered breathing in paediatric patients. In cases of substantial tonsillar hypertrophy or recurrent tonsillitis, it is generally performed with a tonsillectomy. This procedure may be indicated for disorders such as rhinosinusitis, reduced olfactory function, and suspicious neoplasms [12]. The 2022 guidelines for tympanostomy tube insertion recommend the procedure for children with bilateral chronic OME and documented hearing loss, or those with unilateral/bilateral OME and related symptoms (e.g., balance issues). It is also indicated for recurrent AOM with effusion and for at-risk children with persistent OME [3].

A recent study has shown that inflammatory markers in the blood, specifically the neutrophil-lymphocyte ratio (NLR) and mean platelet volume (MPV), may assist in distinguishing between serous and mucoid types of otitis media with effusion (OME), especially in children suffering from obstructive adenoid hypertrophy (OAH) [13]. Yükkaldıran et al. observed that increased NLR and platelet-lymphocyte ratio (PLR) were linked to the development of OME and showed a correlation with hearing thresholds, indicating that these measures might aid in the diagnosis and evaluation of hearing conditions [2]. While rare in adults, OME is extremely common in young children, making early identification strategies critical in paediatric practice [14]. OME is now increasingly recognised as a multifactorial disease, with inflammation, epithelial changes, and effusion properties all contributing to its chronicity and recurrence [15]. Furthermore, the degree of hearing loss often corresponds to the type of middle ear fluid, with mucoid effusions typically producing more severe impairment than serous types [14]. However, although this research highlights the potential diagnostic significance of inflammatory markers, it did not differentiate between the types of effusion (serous vs. mucoid) or investigate how these markers could influence the decision to undertake surgical intervention. The presence of NET DNA and mucins enhances fluid viscosity, supports biofilm persistence, and maintains local inflammation. Neutrophilia and elevated NLR/SII indicate a more pronounced neutrophil-dominant response, which likely correlates with poorer or delayed hearing recovery post-surgery [16,17]. Our study sought to address this limitation by assessing the connection between inflammatory markers, the nature of the effusion, and audiological results, ultimately aiming to enhance treatment choices and possibly decrease unnecessary surgical procedures. A first objective was to determine whether certain blood inflammatory markers (e.g., NLR, PLR, EBR, SII, and MPV) can pre-operatively predict effusion characteristics. A second objective was to assess the relationship between these inflammatory markers and post-operative hearing improvement in children undergoing adenoidectomy with tympanostomy tube insertion, and to determine whether blood inflammatory markers can facilitate surgical decision-making.

## 2. Materials and Methods

### 2.1. Study Design and Setting

This is a retrospective cohort study carried out in a tertiary care government hospital in Al-Ahsa, KSA. Paediatric patients who had undergone adenoidectomy, with or without tympanostomy tube (TT) placement, were identified between 1 January and 31 December 2024. The study was reviewed and approved by the King Faisal University Faculty of Medicine Research Ethics Committee (Ref. KFU–REC–2025–APR–ETHICS3279). The need for informed consent was waived because the research was retrospective and anonymised. The study adhered to the STROBE (Strengthening the Reporting of Observational Studies in Epidemiology) guidelines for reporting observational research (Supplementary Material). 

### 2.2. Inclusion and Exclusion Criteria

All children under 12 years of age who had undergone adenoidectomy for OAH, with or without TT insertion, were evaluated during the study year. The diagnosis of OME was made based on clinical, tympanometric, and/or radiological examination findings in the patient records. An individual surgeon made the determination of the type of effusion by using direct visual assessment during the surgery. This determination was based on the nature and aspect of the fluid that was present in the middle ear, and it was characterised as either serous or mucoid. Serous effusions had a thin, clear texture, while mucoid effusions had a thick, pus-filled look. There was no grading scale or photographic documentation used during the operation.

The non-effusion group consisted of children undergoing adenoidectomy without the insertion of tympanostomy tubes. Before the surgery, a standardised diagnostic approach that included a clinical history, pneumatic otoscopy, and tympanometry revealed that the patient did not have otitis media with effusion (OME). Children did not show any symptoms connected to OME, such as hearing loss, speech delay, or recurring infections.

Pneumatic otoscopy showed that the tympanic membrane moved normally without bulging or fluid, and tympanometry showed type A curves, which indicated that the middle ear pressure and compliance were normal. Age-appropriate pure-tone audiometry was subsequently used to confirm normal hearing thresholds. This diagnostic protocol is in accordance with current clinical practice guidelines, which suggest pneumatic otoscopy as the initial diagnostic test for OME and tympanometry as a confirmatory adjunctive diagnostic method [10,18].

Patients with no preoperative lab results, missing intraoperative documentation on the type of effusion, or lack of pre- or postoperative audiometry were also excluded. The sample consisted of all eligible patients in the study period, without pre-selection or randomisation.

### 2.3. Data Collection

A data extraction form was designed and reviewed by the research team to achieve consistency in the extraction of the data. The lead author had access to the electronic medical records, and he collected the relevant clinical, laboratory, and audiometric information. To guarantee the precision and reliability of the data, another member of the research group cross-checked all the extracted data against the original records.

Data were collected on patient demographics (age and sex), type of surgical procedure, and preoperative blood investigations. Inflammatory markers were obtained from venous blood samples collected 1–3 days prior to surgery under standard aseptic conditions. Samples were processed in our institutional haematology laboratory using an automated haematology analyser according to standardised protocols. Laboratory parameters were neutrophil, lymphocyte, platelet, eosinophil, basophil, and McPV levels. The formulae used for the calculated indices were the NLR, PLR, eosinophil-to-basophil ratio (EBR), and the systemic immune-inflammation index (SII), which is formulated as (neutrophil × platelet)/lymphocyte.

Age-appropriate methods were used to test hearing. Routine pure-tone audiometry was used for participants aged 6 years or older, and conditioned play audiometry was used for younger children. Hearing thresholds were assessed in decibels hearing level (dB HL), and defined as mild (25–40 dB HL) and moderate (41–55 dB HL). Evaluations were performed preoperatively, and at two weeks and one month postoperatively. Hearing results were categorised as improved (normal hearing regained), persistent mild loss, or deteriorated. The audiologists performing the assessments were blinded to intraoperative findings and blood data.

### 2.4. Statistical Analysis

IBM SPSS Statistics version 28.0 (IBM Corp., Armonk, NY, USA) was used for all statistical analyses. The Shapiro–Wilk and Kolmogorov–Smirnov tests were used to evaluate the normality of distribution for continuous variables. For normally distributed continuous variables, the mean ± standard deviation (SD) was used, and for non-normally distributed variables, the median and interquartile range (IQR) were used. Frequencies and percentages were used to display categorical variables. When necessary, Wilcoxon signed-rank tests or paired t-tests were used to compare the hearing thresholds before and after surgery. For continuous variables, independent t-tests or Mann–Whitney U tests were used to analyse group comparisons based on hearing outcomes. For categorical data, chi-square or Fisher’s exact tests were used. When applicable, odds ratios (ORs) with 95% confidence intervals (CIs) were computed. Statistical significance was defined by a *p* value < 0.05. Patients with incomplete audiometric or laboratory data were excluded from the analysis. No imputation or sensitivity analyses were performed.

## 3. Results

A total of 232 children (113 males and 119 females) undergoing surgery for OAH were included in the study. The children were categorised as Group 1 (*n* = 42) with intraoperatively confirmed serous OME, Group 2 (*n* = 78) with mucoid effusion, or a non-effusion group “Group 3” (*n* = 112) without OME. The mean age of the serous, mucoid, and non-effusion groups was 5.76 ± 2.00, 6.17 ± 2.28, and 5.14 ± 1.95 years, respectively (*p* = 0.008). Regarding gender distribution across the groups, the proportion of males was 50.0%, 52.6%, and 45.5%, respectively (*p* = 0.267). The distribution of blood inflammatory parameters by group is given in Table 1.

The mucoid group exhibited significantly higher neutrophil counts, lymphocyte counts, platelet counts, and SII values compared to the non-effusion group (*p* < 0.001, *p* = 0.034, *p* = 0.005, and *p* = 0.010, respectively). Furthermore, when compared to the serous group, the mucoid group showed significantly elevated neutrophil counts, platelet counts, NLR, eosinophil counts, and SII values (*p* < 0.001, *p* < 0.001, *p* = 0.024, *p* = 0.003, and *p* < 0.001, respectively).

Other inflammatory indices, such as MPV, PLR, basophil counts, and EBR, did not differ significantly across the groups. These findings suggest that children with mucoid OME not only experience poorer postoperative hearing outcomes but also exhibit more pronounced systemic inflammation (Table 2).

Among the groups, in terms of surgical intervention, 112 children (48.3%) underwent adenoidectomy alone, and 120 children (51.7%) received adenoidectomy combined with ventilation tube insertion. To compare hearing across the groups, preoperative hearing levels were evaluated in children with serous and mucoid effusions. In the serous group, 83.3% (*n* = 35) of children had mild hearing loss, and 16.7% (*n* = 7) had moderate hearing loss. Similarly, in the mucoid group, 82.1% (*n* = 64) had mild and 17.9% (*n* = 14) had moderate hearing loss. The distribution of preoperative hearing levels did not differ significantly between the groups (χ^2^ = 0.031, *p* = 0.860), and the odds ratio for moderate hearing loss in the serous group compared to the mucoid group was 1.09 (95% CI: 0.40–2.96), indicating no increased risk.

In contrast, postoperative hearing outcomes after two weeks exhibited a significant difference between the groups. All children in the serous group (100%) achieved normal hearing postoperatively, while 15.4% (*n* = 12) of the mucoid group continued to exhibit mild hearing loss, despite intervention. One-month postoperative follow-up assessments showed normal hearing in all affected children in both groups. This difference was statistically significant (χ^2^ = 7.179, *p* = 0.008). The likelihood of enduring postoperative mild hearing loss was around 16 times greater in the mucoid group than in the serous group (adjusted OR = 15.97, 95% CI not displayed), indicating that mucoid effusions correlate with inferior auditory recovery post-surgery (Table 3).

Only the neutrophil count was significantly enhanced and correlated with mild postoperative hearing loss (*p* = 0.021), suggesting that increased neutrophil counts may act as a possible indicator for negative auditory outcomes following adenoidectomy and tympanostomy tube insertion in paediatric patients. The remaining variables showed no significant differences (Table 4).

## 4. Discussion

This research enhances our understanding of the correlation between inflammatory blood markers and the different subtypes of OME, as well as the postoperative hearing results in children with OAH undergoing surgical treatment. The results align with prior studies emphasising the role of chronic inflammation in the pathogenesis and persistence of mucoid effusions. For instance, mucoid OME has been associated with prolonged inflammatory responses and higher cytokine levels in middle ear effusions, which are thought to delay mucosal healing and auditory recovery following surgery [19]. Similarly, research by Portmann categorised mucoid effusions as part of a more advanced disease continuum, often requiring more time to resolve, even after ventilation tube placement [20].

Our results reveal significantly higher neutrophil, lymphocyte, and platelet counts, as well as elevated SII and NLR values in children with mucoid effusion compared to those in both the serous and non-effusion groups. Eosinophil counts were also significantly increased in the mucoid group, while no significant differences were found for mean platelet volume (MPV), PLR, basophil count, or eosinophil-to-basophil ratio (EBR).

These results are somewhat consistent with prior literature while also highlighting significant differences. A 2025 study with 215 children found no significant changes between groups in SII, PLR, or EBR. However, NLR was considerably lower in the serous group than in the non-effusion group (*p* = 0.023) [13]. Conversely, our study revealed no significant difference in NLR between the serous and non-effusion groups; however, there was a markedly higher NLR in the mucoid group compared to the serous group, indicating that mucoid effusions may signify a more severe or chronic inflammatory condition not present in serous cases.

An earlier study from 2017 involving 250 patients reported that NLR and PLR were significantly higher in the serous group than in the control, but not in the mucoid group, a result that contrasts with our finding that the mucoid group showed consistently higher levels of inflammatory markers [21]. This discrepancy may be due to differences in sample categorisation or criteria for effusion classification.

More closely aligned with our findings are those of a 2018 study which included 47 children with mucoid effusion and 37 with serous effusion [22]. Significantly higher NLR was found in the mucoid group compared to both the serous and non-effusion groups (*p* = 0.001), although PLR did not differ significantly between mucoid and serous cases. Our investigation corroborated considerably increased NLR in mucoid patients and did not identify PLR as a differentiating marker, so validating the hypothesis that NLR may be more sensitive than PLR for detecting systemic inflammation in mucoid OME. A 2015 study with 154 patients corroborates several of our findings [23]. Significantly increased white blood cell, neutrophil, and lymphocyte counts were reported in the mucoid OME group compared to a non-effusion group, and notably higher lymphocyte counts were detected in the mucoid group compared to the serous group, corroborating our own lymphocyte findings. This study revealed greater platelet counts in serous instances than in mucoid cases (*p* = 0.004), whereas our data indicated higher platelet counts in the mucoid group. This could be because the two studies were looking at different stages of inflammation or for other reasons.

This study identified elevated neutrophil count as the sole significant blood inflammatory marker associated with mild postoperative hearing loss after adenoidectomy and tympanostomy tube insertion (*p* = 0.021), suggesting its potential as a predictive factor for less favourable immediate auditory recovery. This finding contrasts with a 2020 retrospective study involving 244 paediatric OME patients, which reported significant differences in lymphocyte counts (increasing with hearing loss severity), and inverse relationships with NLR and PLR (decreasing with severity), as well as negative correlations between hearing levels and NLR, PLR, and MPV [2]. This discrepancy may indicate complex and varied inflammatory responses in OME. The existing literature indicates correlations between white blood cells, particularly neutrophils, and various forms of hearing impairments, including sudden sensorineural hearing loss (SSHL), so underscoring the systemic relationship between inflammatory processes and auditory function [24]. Preoperative neutrophil count and associated inflammatory indices, including the neutrophil-to-lymphocyte ratio (NLR) and systemic immune-inflammation index (SII), can function as additional indicators for risk classification. These markers could [17] identify patients at increased risk of delayed recovery who would benefit from expedited surgery or enhanced audiologic monitoring, and [2] select subjects for trials of targeted anti-inflammatory interventions [16,17]. It is essential that these biomarkers be integrated with clinical variables and validated prospectively prior to their application in individual patient decision-making [2,16,17]

OME’s classification into mucoid and serous variants is of substantial clinical and prognostic significance. Serous effusions usually reflect acute or resolving situations with reduced inflammatory burden, whereas mucoid effusions, which are characterised by increased viscosity and elevated protein concentration, usually result from protracted or recurrent inflammation [25,26]. These biological distinctions affect variability in illness progression, responsiveness to tympanostomy tube insertion, and the degree of postoperative auditory improvement. Research suggests that children with mucoid otitis media with effusion are at an increased risk of developing auditory impairments and often experience delayed mucosal recovery, even after receiving appropriate surgical treatment [27]. Thus, accurately identifying the effusion subtype during surgery or through further diagnostics (e.g., tympanometry, middle ear fluid analysis) might aid doctors in evaluating risk and customising postoperative treatment methods [28].

The management of OME is complex, and due to numerous guidelines and studies, a consensus on treatment methods remains elusive. There has been an attempt to manage the disease with steroids, antibiotics, antihistamines, decongestants, and even autoinflation treatment. However, even with all these interventions, OME is often refractory and the patient’s hearing does not improve [29]. The United States was the first country to use balloon dilation Eustachian tuboplasty (BDET) in the year 2010, and this procedure was developed to address Eustachian tube dysfunction (ETD) [30] A research has indicated that balloon Eustachian tuboplasty may serve as an effective primary intervention for Eustachian tube dysfunction in children, providing a possible replacement for gold standard surgical options. Nevertheless, additional studies are required to assess its efficacy over a protracted duration [31].

The research did not include recent infections, allergies, or environmental exposures like smoke as potential influences on inflammation marker levels. Moreover, blood inflammatory markers are subject to diurnal variation, stress, and even minor infections. Future studies should address these potential reliability issues by incorporating additional measurements. Follow-up studies of longer duration would provide more data on whether inflammation markers can effectively inform on lasting hearing recovery or recurrence. The classification of effusion (Serous Vs. Mucoid) in this study relied on the intraoperative assessment by the operating surgeon, which is inherently subjective and may lead to observer bias in classification. Subsequent research should implement blinded assessments and standardised objective measures, such as rheometry, mucin quantification, or blinded photographic evaluations, to minimise misclassification.

## 5. Conclusions

Our retrospective cohort study indicates that children with mucoid otitis media with effusion (OME) demonstrate elevated systemic inflammation, as evidenced by increased neutrophil counts, neutrophil-to-lymphocyte ratio (NLR), and systemic immune–inflammation index (SII), compared to those with serous effusion. Among the preoperative markers analysed, only the absolute neutrophil count demonstrated a statistically significant association with a minor yet persistent postoperative hearing deficit. This retrospective analysis may be subject to unmeasured confounding and variability in perioperative care; thus, the findings should be viewed as hypothesis-generating rather than confirmatory. The findings indicate that preoperative neutrophil count could serve as a predictor for children at risk of delayed auditory recovery post surgery. However, further prospective and standardised studies are necessary to validate this association and to determine if including neutrophil count in preoperative evaluations enhances clinical decision-making and patient outcomes.

## Figures and Tables

**Table 1:** The distribution of blood inflammatory parameters by group.

Variable	Group		
	Serous	Mucoid	Control
	Mean (SD)	Mean (SD)	Mean (SD)
Age	5.76 (2.00)	6.17 (2.28)	5.14 (1.95)
Neutrophil count (10^3^/μL)	3.66 (2.04)	5.33 (2.10)	4.12 (1.85)
Lymphocyte count (10^3^/μL)	2.70 (1.08)	3.07 (0.98)	2.69 (0.94)
Platelet count (10^3^/μL)	255.21 (79.37)	321.44 (89.77)	277.95 (86.24)
Mean Platelet Volume	8.87 (1.33)	9.36 (1.30)	9.25 (1.36)
Neutrophil–Lymphocyte Ratio	1.59 (1.16)	1.97 (1.08)	1.73 (1.01)
Platelet–Lymphocyte Ratio	112.47 (64.17)	120.00 (64.75)	117.41 (59.69)
Eosinophil count (10^3^/μL)	174.40 (108.45)	243.53 (372.90)	154.42 (83.02)
Basophil count (10^3^/μL)	1.67 (1.31)	1.96 (1.00)	1.66 (0.89)
Eosinophil–Basophil Ratio	331.46 (487.54)	387.67 (813.49)	248.78 (518.69)
Systemic Immune Index	419.59 (370.71)	632.34 (382.67)	477.18 (329.07)

**Table 2:** Comparison of inflammatory parameters between groups by the Mann–Whitney U test.

Group	Neut	Lym	Plt.C	MPV	NLR	PLR	E	B	EBR	SII
Serous–Control	0.348	1.000	0.472	0.373	0.437	1.000	1.000	1.000	1.000	0.268
Serous–Mucoid	<0.001	0.132	<0.001	0.141	0.024	0.968	0.003	0.186	0.329	<0.001
Mucoid–Control	<0.001	0.034	0.005	1.000	0.290	1.000	0.214	0.114	0.967	0.010

Neut, neutrophils; Lym, lymphocytes; Plt.C, platelet count; MPV, mean platelet volume; NLR, neutrophil-to-lymphocyte ratio; PLR, platelet-to-lymphocyte ratio; E, eosinophils; B, basophils; EBR, eosinophil-to-basophil ratio; SII: systemic immune index.

**Table 3:** Hearing assessment (unadjusted odds ratios with 95% confidence intervals).

**Preoperative Hearing (dB)**						
Group	Variable	Mild	Moderate	Total	OR (95% CI)	*p* value
	Serous	35	7	42	1.094	0.537
	Mucoid	64	14	78		
Postoperative hearing (dB)						
Group	Variable	Normal	Mild	Total	15.97	0.004
	Serous	42	0	42		
	Mucoid	66	12	78		

Note: OR = odds ratio; CI = confidence interval. Odds ratios are unadjusted.

**Table 4:** Assessment of the relationship between inflammatory blood markers and postoperative hearing status in children undergoing adenoidectomy with tympanostomy tube insertion.

Variable	Postoperative Hearing (dB)				*p* Value
	Normal		Mild		
	Mean	SD	Mean	SD	
Age	5.97	2.23	6.50	1.83	0.373
Neutrophil count (10^3^/μL)	4.58	2.12	6.30	2.68	0.021
Lymphocyte count (10^3^/μL)	2.94	1.05	2.97	0.93	0.856
Platelet count (10^3^/μL)	296.32	94.85	312.75	60.86	0.537
Mean Platelet Volume	9.15	1.29	9.61	1.64	0.304
Neutrophil–Lymphocyte Ratio	1.77	1.07	2.45	1.42	0.097
Platelet–Lymphocyte Ratio	117.79	67.01	113.83	39.29	0.603
Eosinophil count (10^3^/μL)	219.58	324.66	207.92	110.07	0.584
Basophil count (10^3^/μL)	1.79	1.08	2.35	1.41	0.090
Eosinophil–Basophil Ratio	354.63	684.54	508.68	990.57	0.551
Systemic Immune Index	537.31	382.90	747.75	439.10	0.078

## Data Availability

The data presented in this study are available upon reasonable request from the corresponding author. The data are not publicly available due to privacy or ethical restrictions.

## Supplementary Materials

The following supporting information can be downloaded at: https://www.mdpi.com/article/10.3390/medicina61091520/s1, Table S1: STROBE (Strengthening the Reporting of Observational Studies in Epidemiology) guidelines for reporting observational research.
